# Synthesis and Characterization of Nanostructured Oxide Layers on Ti-Nb-Zr-Ta and Ti-Nb-Zr-Fe Biomedical Alloys

**DOI:** 10.3390/jfb14040180

**Published:** 2023-03-24

**Authors:** Gabriela Strnad, Laszlo Jakab-Farkas, Federico Simone Gobber, Ildiko Peter

**Affiliations:** 1 Department of Engineering and Industrial Management, Faculty of Engineering and Information Technology, “GE Palade” University of Medicine, Pharmacy, Science and Technology of Targu Mures, 540139 Targu Mures, Romania; 2 Department of Mechanical Engineering, Faculty of Technical and Human Sciences, Sapientia University of Cluj Napoca, 540485 Targu Mures, Romania; 3 Department of Applied Science and Technology, Politecnico di Torino, 15121 Alessandria, Italy

**Keywords:** electrochemical anodization, TiO_2_ nanopores/nanotubes, Ti-Nb-Zr-Ta alloys, Ti-Nb-Zr-Fe alloys

## Abstract

Nanoporous/nanotubular complex oxide layers were developed on high-fraction β phase quaternary Ti-Nb-Zr-Ta and Ti-Nb-Zr-Fe promising biomedical alloys with a low elasticity modulus. Surface modification was achieved by electrochemical anodization aimed at the synthesis of the morphology of the nanostructures, which exhibited inner diameters of 15–100 nm. SEM, EDS, XRD, and current evolution analyses were performed for the characterization of the oxide layers. By optimizing the process parameters of electrochemical anodization, complex oxide layers with pore/tube openings of 18–92 nm on Ti-10Nb-10Zr-5Ta, 19–89 nm on Ti-20Nb-20Zr-4Ta, and 17–72 nm on Ti-29.3Nb-13.6Zr-1.9Fe alloys were synthesized using 1 M H_3_PO_4_ + 0.5 wt% HF aqueous electrolytes and 0.5 wt% NH_4_F + 2 wt% H_2_0 + ethylene glycol organic electrolytes.

## 1. Introduction

Titanium-based materials are extensively used for biomedical implants in dental and orthopedic applications due to their excellent biocompatibility, osseointegration capacity, and good mechanical properties. A recent research direction has been the development of titanium alloys that contain a large amount of β phase; by this, the elasticity modulus can be lowered close to that of human cortical bone (E = 15–30 GPa) in order to avoid a stress shielding effect.

Currently, Ti-based alloys such as Ti-Nb-Zr-Ta- and/or Ti-Nb-Zr-Fe-based alloys are considered to be multifunctional alloys because they are commonly employed in various fields. As reported in a few studies, these applications are oriented to several engineering applications—in particular, aerospace and automotive [1,2,3,4] and agricultural applications [5]—and evidently also includes the biomedical field, where bone and dental implants are usually developed with such alloys [6,7]. As for biomedical application concerns, these alloys are remarkable because of their safe compositions with no significant toxic effects on the human body. These alloys are good candidates to progressively replace other metallic alloys [8,9,10,11,12].

Biocompatibility is the result of the inertness of titanium alloys based on the spontaneous formation of a stable, protective, and strongly adherent thin titanium oxide with a thickness in the nanometer range on the material surface. The osseointegration of titanium-based implants can be further enhanced by a modification of their surface morphology, and an important goal in implant design is to mimic the unique micro- and nanoscale characteristics of bone [13,14,15]. At a microscale level, the modification aims to develop microrough morphologies with an increased bone-to-implant contact area, whereas nanoscale modifications intend the synthesis of a nanostructured oxide morphology (nanopores and nanotubes). Studies show that cells cultured on nanostructured surfaces exhibit a higher adhesion, proliferation, ALP activity, and bone matrix deposition compared with those grown on flat titanium surfaces [16,17,18]. Increased blood serum protein adsorption, platelet adhesion and activation [19], and enhanced antibacterial properties [20,21], demonstrated in vitro and in vivo [21,22], have been reported regarding modified nanoporous/nanotubular titania (TiO_2_) surfaces. The favorable osseointegration around TiO_2_ nanopores/nanotubes of modified surfaces of 15–100 nm diameter has been demonstrated by the overexpression of genes related to the high activity of osteoblasts and mesenchymal stem cells, and by fast kinetics of hydroxyapatite formations surrounding titanium implants [23,24,25,26]. Furthermore, these nanostructured surfaces are of particular interest for bioactive implants with functional surfaces to perform as carriers for the delivery of drugs and growth factors as well as anti-inflammatory, antibacterial, and anticancer agents [27,28,29,30].

The modification of the surface oxide layer can be effectively and reliably carried out by electrochemical anodization when using proper electrolyte compositions and process parameters. If the goal is to develop a nanotubular/nanoporous oxide, there are several types of electrolytes that promote these morphologies, including inorganic aqueous electrolytes, organic electrolytes containing F ions, and non-fluoride-based electrolytes. The mechanism of the growing of nanotubular/nanoporous oxides is explained by the field-assisted dissolution model as being based on the existence of fluoride ions and on the equilibrium conditions of oxide formation at the metal–oxide interface vs. oxide etching at the oxide–electrolyte interface [31,32]. The oxide grows similar to a digging process, from the top down, to form pore channels. Lately, there have been reports that bring evidence against this model [33,34,35] and propose that a layer of metastable titanium-oxyfluorides at the oxide/metal interface that flows up, forming the neighboring pore wall upon pore growth, is the key for the formation of titania nanotubes [36]. A new explanation of oxide development is also based on the observation that nanotubular morphologies can be synthesized even in the absence of fluoride ions, which are critical for the etching phase, suggesting that the oxide-growing mechanism model relies on electronic current theory and the oxygen bubble mold effect, where the oxide layer grows from the bottom up around the oxygen bubbles [37]. Currently, more research is needed in order to fully understand the unclear growth mechanism of nanoporous/nanotubular oxide layers developed by electrochemical anodization and even more so on complex quaternary titanium alloys, which are studied in present work.

The synthesis of nanostructured TiO_2_ layers is well-reported in the literature, especially on samples of the foil-type, which are planar and highly polished. In practice, implant surfaces are expected to be of complex shapes with a microrough topography, depending on the particularities of the manufacturing process. The development of nanoporous/nanotubular TiO_2_ layers with pore/tube openings in the range of 20–140 nm on pure Ti and ternary Ti6Al4V alloys on planar, cylindrical, and screw-type surface geometries [38,39], having an initial surface processed by different types of technologies (turning, grinding, sand blasting, polishing, and chemical etching [40]) has been successfully carried out by us in the last years, allowing us to accumulate the technological knowledge to advance to the electrochemical anodization of complex quaternary titanium alloys.

Regarding the electrochemical anodization of complex alloys in the Ti-Nb-Zr system, depending on the specific type of alloy, both α and β phases are present with the addition of the new elements. It is expected that organized nanotubular complex oxides will form during the α phase, whereas a combination of nanotubes and nanopores will grow during the two-phase (α + β) component [41]. Several studies have reported on the surface modification of ternary Ti-30Nb-xZr [42]; Ti-35Nb-5Zr, Ti-35Nb-10Zr, and Ti-35Nb-15Zr [43]; Ti-35Nb-2Zr and Ti-35Nb-4Zr [44]; and Ti-13Nb-13Zr [45,46]. This has brought valuable knowledge on important process parameter choices. Furthermore, a delicate electrochemical anodization process for the preparation of quaternary alloys Ti-Nb-Zr-Sn [47] and Ti–Nb–Ta–Zr [48] was reported to be successful for the synthesis of nanostructured oxide layers in specific technological conditions. Regarding iron-containing titanium alloys, very recently, Rios et al. reported the anodization of Ti-30Nb-3Fe and Ti-30Nb-5Fe, resulting in a nanostructured layer with a bimodal size distribution where the large nanotubes were surrounded by small ones [49].

In this context, the present research aimed to study the synthesis of nanoporous/nanotubular oxide layers on the surface of newly developed β phase-containing quaternary titanium alloys Ti-10Nb-10Zr-5Ta, Ti-20Nb-20Zr-4Ta, and Ti-29.3Nb-13.6Zr-1.9Fe [12] with an initial microrough topography, and to discover proper electrochemical anodization process parameters to be used when working with aqueous and organic electrolytes.

## 2. Materials and Methods

Ti-10Nb-10Zr-5Ta, Ti-20Nb-20Zr-4Ta, and Ti-29.3Nb-13.6Zr-1.9Fe titanium alloys of high-fraction β-type were synthesized from elemental components by melting in a cold crucible furnace by levitation. Their synthesis as well as their structural, compositional, and mechanical characterization have been reported elsewhere [12]. The alloys were cast as cylinders of approximately 18.5 mm in diameter. The samples that were subjected to surface modification were in the form of a disc of 3 mm thickness cut from the cast cylinders. The planar surfaces of the disc samples were ground with 320, 600, and 1200 grit SiC papers (ATM). After each grinding phase, the samples were carefully rinsed in deionized water and ethanol, cleaned using a 3.0 Dr. Mayer (Dr. Mayer Life & Health, Regensburg, Germany) ultrasonic cleaning machine, and dried in hot air using a BOV-T25F (Biobase, Wolfenbüttel, Germany) drying unit. By this preparation process, a microrough-type morphology of the planar surfaces of the disc samples was provided, with a roughness Ra of 0.2–0.5 µm, measured using an SJ-310 (Mitutoyo, Kanagawa, Japan) roughness tester. For the further modification of the surface, the as-prepared samples were subjected to electrochemical anodization (EA).

The laboratory setup for the electrochemical anodization of the samples was a two-electrode-type. For each deposition experiment, the sample was placed at the anode; the cathode was a disc 20 mm in diameter made of pure copper. The experiments were performed using two types of electrolyte, an aqueous electrolyte (1 M H_3_PO_4_ + 0.5 wt% HF) and an organic electrolyte (0.5 wt% NH_4_F + 2 wt% H_2_0 + ethylene glycol). The volume of the electrolyte was 60 mL and the distance between the anode and cathode was 20 mm. The current was supplied by a programmable dual-range DC power supply (9184B; BK Precision). Experiments in the aqueous electrolyte were performed at an anodization potential of U = 20 V, applied with an initial ramp of U_r_ = 0.2 V/s for a duration of T = 30 min. When using the organic electrolyte, the anodization potentials were U = 20, 40, and 60 V; U_r_ = 1 V/s and the durations were T = 10 and 30 min. Nanosource2 software designed by us was used to control the anodization experiments and to monitor and register the process parameters. After anodization, the samples were rinsed in deionized water, cleaned in ethanol, and dried in air. An AEA-100G (Adam Equipment, Milton Keynes, UK) analytical balance was used for the evaluation of the mass changes of the samples.

For the optical microscopy investigations, the samples were etched using a 5% HF + 15% HNO_3_ + 80% H_2_O solution with a holding time of 10 s in the etching solution, then washed in water and dried. The metallographic observation was carried out directly after the chemical attack using an optical microscope (OM, Olympus BX51M type, Olympus Corporation, Tokyo, Japan). The morphological characterization was carried out using a scanning electron microscope operated at 25 kV (JSM 5200 (JEOL, Tokyo, Japan) and Evo 15 (Zeiss, Jena, Germany)). The nanostructure dimensions were measured and evaluated using open source ImageJ software 1.46r, and the statistical data and plots were processed using Origin software 10.0.0.154 (OriginLab, Northampton, MA, USA). The distribution of the elements was verified by energy-dispersive X-ray spectrometry (EDS, Ultim Max, Oxford Instruments, Abingdon, UK). The phase analysis was performed using the X-ray diffraction technique (X-ray; PANanalytical tool) using Cu Kα radiation at 40 kV and 40 mA with a scanning rate of 2 (°)·min^−1^.

## 3. Results and Discussion

The research carried out in this study began from the outcomes obtained from a previous study, reported in [12]. Here, we mentioned an important aspect; in particular, the phase composition of the titanium alloys developed and now subjected to electrochemical anodization in order to develop the nanostructured surfaces:

- The Ti-10Nb-10Zr-5Ta alloy contained 55% α-Ti phase and 45% β-Ti phase;
- The Ti-20Nb-20Zr-4Ta alloy contained 15% α-Ti phase and 85% β-Ti phase;
- The Ti-29.3Nb-13.6Zr-1.9Fe alloy contained 100% β-Ti phase.

From [12], the alloys exhibited good mechanical properties. The ultimate tensile strength (UTS) = 700–1000 MPa, the yield strength (YS) = 330–580 MPa, and—most importantly—had a low elastic modulus of E = 40–50 GPa, which is close to that of human bone (E = 15–30 GPa).

**Figure 1:**
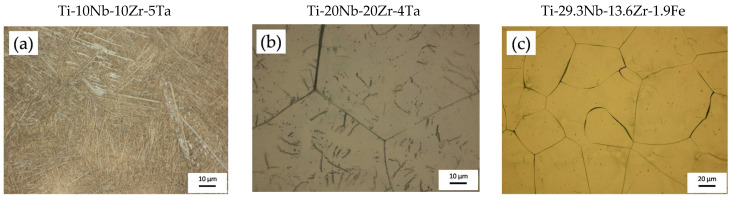
Optical micrographs showing the microstructure of titanium alloys prior to electrochemical anodization: (**a**) Ti-10Nb-10Zr-5Ta alloy; (**b**) Ti-20Nb-20Zr-4Ta alloy; (**c**) Ti-29.3Nb-13.6Zr-1.9 Fe alloy.

Figure 1 shows optical micrographs of the microstructure of the quaternary titanium alloys as a result of casting after the melting of the components in a cold crucible furnace by levitation. Ti-10Nb-10Zr-5Ta had a lamellar structure, whereas Ti-20Nb-20Zr-4T and Ti-29.3Nb-13.6Zr-1.9 Fe presented polygonal equiaxed grains. Samples with these initial microstructures were prepared and the electrochemical anodization was performed as stated above, using aqueous and organic electrolytes and different process parameters in order to fine-tune the process to enable the effective and reliable synthesis of nanoporous/nanotubular oxide layers with nanostructure inner diameters of 15–100 nm.

### 3.1. Surface Morphology and Topography Evaluation after Electrochemical Anodization

**Figure 2:**
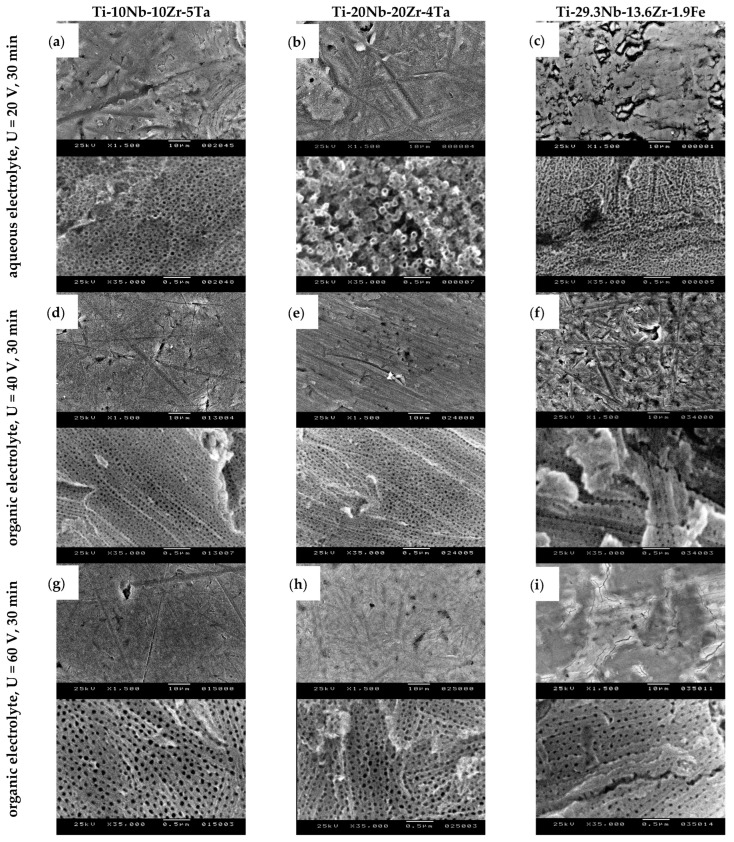
SEM micrographs showing the nanoporous/nanotubular morphology of oxide layers developed by electrochemical anodization on quaternary titanium alloys in aqueous and organic electrolytes: (**a**,**d**,**g**) Ti-10Nb-10Zr-5Ta alloy; (**b**,**e**,**h**) Ti-20Nb-20Zr-4Ta alloy; (**c**,**f**,**i**) Ti-29.3Nb-13.6Zr-1.9 Fe alloy (low magnification ×1500, up; high magnification ×35,000, down).

**Figure 3:**
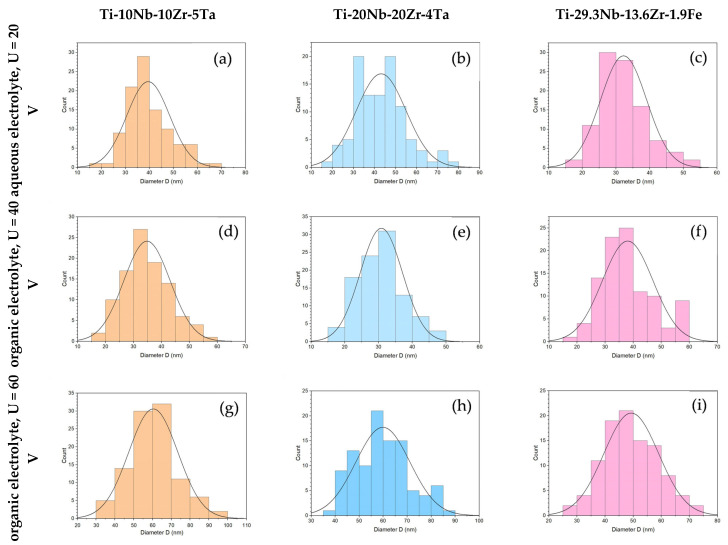
Histograms showing the distribution of the diameters of the nanopores/nanotubes, depending on the EA process parameters: (**a**,**d**,**g**) Ti-10Nb-10Zr-5Ta alloy; (**b**,**e**,**h**) Ti-20Nb-20Zr-4Ta alloy; (**c**,**f**,**i**) Ti-29.3Nb-13.6Zr-1.9 Fe alloy.

Figure 2 shows SEM top-view micrographs taken at different magnifications (low magnification ×1500, up; high magnification ×35,000, down), revealing the nanostructured oxide layers that had developed on the anodized titanium alloys by using different electrolytes and process parameters. The nanostructure dimensions were measured and processed and the results were generated from 100 nanostructures from different positions for each sample. Table 1 presents the results of the important characteristic values and Figure 3 presents the distribution of the openings of the nanopores/nanotubes.

Th surface morphology and the topography evaluation of the samples anodized in the aqueous electrolyte showed that on the Ti-10Nb-10Zr-5Ta alloy, a nanotubular layer was formed; the nanotubes had a regular shape with a compacted and ordered structure. An analysis of the dimensions of the nanofeatures revealed nanotube openings of 39 ± 8.9 nm (average ± standard deviation); the oxide nanotube diameters had a range of 20–69 nm (minimum–maximum) and the most frequent value (mode) was 38 nm (Table 1). Figure 3a shows the distribution of the diameter of the nanotubes, with the most frequent values in the range of 30–45 nm. The anodization of Ti-20Nb-20Zr-4Ta in the same process conditions also led to a nanostructured morphology of the oxide layer. The SEM micrograph (Figure 2b) showed that separated nanotubes were developed. Their diameter of 43 ± 11.8 nm was slightly larger than in the case of Ti-10Nb-10Zr-5Ta; the most frequent values were 30–55 nm (Table 1 and Figure 3b). It was interesting to note the bimodal appearance of the nanotubes; the larger ones were surrounded by smaller ones, an observation that was consistent with recently reported nanostructured oxide layers developed on Ti-35Nb-2Zr [44] and Ti-30Nb-5Fe [49] alloys in an aqueous electrolyte containing 0.3% HF. Regarding the Ti-29.3Nb-13.6Zr-1.9Fe β phase alloy, Figure 2c shows the synthesis of a nanoporous layer. The pore openings were 32 ± 6.8 nm in the range of 17–52 nm, and the most frequent values were in the 25–35 nm range (Table 1 and Figure 3c). The mass of the samples suffered a minimal loss of 1–1.5 mg (0.016–0.018% from the initial mass) due to the effect of anodic dissolution, where metal ions migrated from the surface to the electrolyte solution.

Experiments in the organic electrolyte were begun by using the anodization potential of 20 V, which provided us with good results in previous research works where we modified the surface of a Ti6Al4V alloy and developed a nanotubular/nanoporous oxide layer [38,40]. This was also reported as being successful in the nanotubular oxide morphology development of a Ti-13Nb-13Zr alloy anodized in an organic electrolyte of 0.86 wt% NH_4_F + 47.14 wt% H_2_O + glycerol [45] and a Ti-24Nb-4Zr-8Sn alloy anodized in 0.25 M NH_4_F + 25 vol% H_2_O + glycerol [47]. The durations of the anodization processes were 10 and 30 min, and led to the development of a compact oxide layer on all 3 alloys. Regarding Ti-10Nb-10Zr-5Ta, the alloy where the α phase amount was higher (55%), pitting of the oxide layer was apparent, indicating the initiation of the growth of nanopores. Regarding the Ti-20Nb-20Zr-4Ta alloy containing mainly β phase (85%) and the Ti-29.3Nb-13.6Zr-1.9Fe β-type alloy, the initiation of nanopores did not occur. Consequently, we increased the anodization potential to 40 V and 60 V in order to develop a targeted nanostructured morphology of oxide layers.

Figure 2d–f show the development of nanostructured oxide layers on the surface of all 3 alloys when the potential was U = 40 V, applied with an initial ramp of U_r_ = 1 V/s and a duration of T = 30 min. Regarding the Ti-10Nb-10Zr-5Ta and Ti-20Nb-20Zr-4Ta alloys, the oxide layers were developed with a homogenous morphology, with nanopores close one to another (Figure 2d,e), presenting an internal diameter of 35 ± 8.3 nm and 31 ± 6.3 nm (Table 1), respectively. The range of the distribution of the diameter values (Figure 3d,e) was narrower compared with the one in the case of anodization in a fluoride-based aqueous electrolyte. Regarding the β phase Ti-29.3Nb-13.6Zr-1.9Fe alloy, a nanoporous morphology was also present (Figure 2f); the average nanopore diameter was slightly larger compared with the other 2 alloys at 38 ± 9.0 nm. This was due to the fact that in addition to the most frequent 30–40 nm pores, the oxide layer contained pores of 55–60 nm formed with a 9% frequency (Figure 3f). The nanopores were placed in a regular manner, forming rows; the distance between them was larger in the case of Ti-29.3Nb-13.6Zr-1.9Fe. The mass loss of the samples was 0.4–0.8 mg (0.009–0.012% from the initial mass), lower than in the case of anodization in the aqueous electrolyte.

The fine-tuning of the anodization process was achieved using the following parameters: anodization potential U = 60 V, applied with an initial ramp of U_r_ = 1 V/s, and a duration of T = 30 min. The top-view SEM micrographs presented in Figure 2g–i demonstrate the ordered and compact nanoporous morphology of the deposited oxide layers. A higher anodization potential led to larger pore diameters on all three alloys. Regarding Ti-10Nb-10Zr-5Ta, the average value of the diameter was 60 ± 13 nm compared with 35 ± 8.3 nm obtained at 40 V (Table 1). Large pores of 70–90 nm were also formed, with a frequency of 20% (Figure 3g). The same observations were also valid for Ti-20Nb-20Zr-4Ta, comparing the 60 ± 11.3 nm diameter and 40–89 nm range (at 60 V) with the 31 ± 6.3 nm diameter and 17–46 nm range (at 40 V) (Figure 2h and Table 1). Regarding β phase Ti-29.3Nb-13.6Zr-1.9Fe, the nanopores were smaller than those formed on the α + β phase alloys; their dimensional characteristics had a 49 ± 9.7 nm diameter in the 25–72 nm range (Figure 2i and Table 1). Compared with the nanopores formed on the same alloy at an anodization potential of 40 V, there was an increase in the pore openings. By increasing the potential, the mass loss also increased to 1.4–3.2 mg (0.015–0.063%) as the electrical field was higher, promoting the migration of positive metallic ions from the anode surface to the electrolyte solution.

### 3.2. EDS Analysis

An EDS analysis was used for an elemental qualitative analysis of the alloys and partially to provide a semi-quantitative interpretation of the results as well.

**Figure 4:**
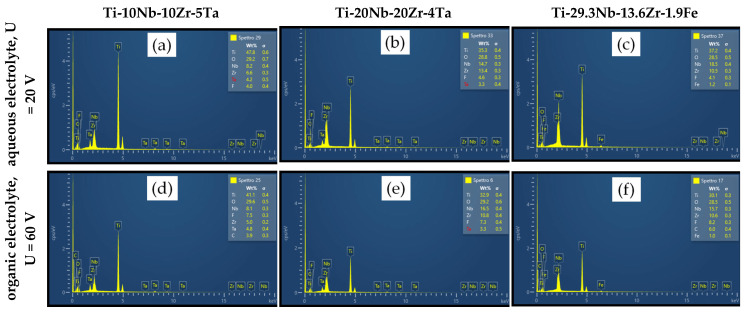
Elemental composition of nanostructured oxide layers developed by EA in aqueous and organic electrolytes: (**a,d**) Ti-10Nb-10Zr-5Ta alloy; (**b**,**e**) Ti-20Nb-20Zr-4Ta alloy; (**c,f**) Ti-29.3Nb-13.6Zr-1.9 Fe.

The EDS spectrum and the elementary chemical composition obtained after the analysis are illustrated in Figure 4a–f and Table 2. As estimated, all alloy compositions revealed a high amount of oxygen, indicating the growth of a complex metal oxide on the sample surfaces. The presence of all alloying elements was confirmed; major elements (Ti, Nb, Zr, Ta and Fe) and O were present in a higher amount whilst the existence of F and C at a minor quantity was due to their uptake from the electrolytes. Based also on their electrochemical properties, Ti and Zr showed a higher tendency to substitute elements from the liquid solution and had more affinity vs. oxygen as well. First, there was a chemical dissolution of Ti (prevalently) and Zr caused by the electrolyte, followed by competition among the O^2−^ ions for the formation of the oxide and F^−^ ions, which also determined the dissolution of the oxide. Nb and Ta had a lower attraction vs. the O^2−^ ions and, therefore, stable oxide formation was delayed in the case of such elements. However, Nb and Ta remained prevalently incorporated in the alloy.

Nanopores that had developed with a different orientation along the surfaces were observed, but their chemical composition obtained from smaller selected areas did not reveal any significant difference compared with the entire area results, as reported in Table 2.

### 3.3. XRD Analysis

**Figure 5:**
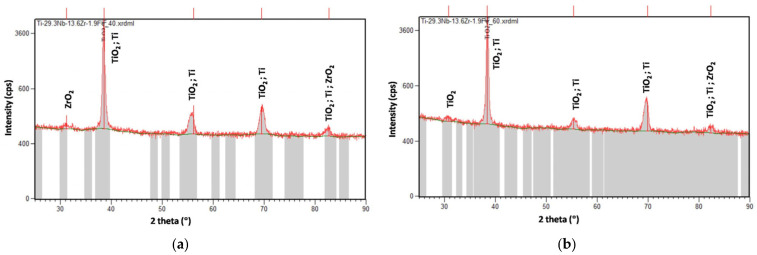
XRD results of nanoporous oxide layers developed on Ti-29.3Nb-13.6Zr-1.9 Fe alloy: (**a**) EA in organic electrolyte, anodization potential 40 V; (**b**) EA in organic electrolyte, anodization potential 60 V.

The XRD analysis revealed that, in all cases, there was an amorphous appearance of the spectra (it mostly contained broader peaks) and the presence of multicomponent oxides, TiO_2_ and ZrO_2_; their development was identified. Depending on the deposition conditions, the intensity of the peaks was slightly different. For all samples, the most stable TiO_2_ form, anatase in a tetragonal crystalline structure and orthorhombic ZrO_2_, was detected. The XRD spectra of the titanium alloy containing Fe are shown in Figure 5.

### 3.4. Current Evolution Analysis

**Figure 6:**
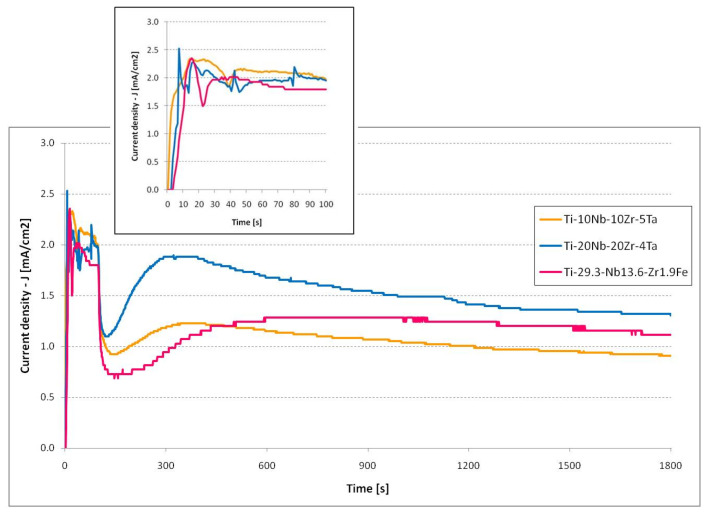
Current evolution during the EA of quaternary titanium alloys in aqueous electrolyte (inset: first 100 s, the potentiodynamic stage).

The current density vs. time plots showed the current evolution during the electrochemical anodization in the aqueous electrolyte (Figure 6), which presented a typical evolution for nanoporous/nanotubular oxide growth. A steady state of current appeared during the formation of these nanostructures, as recognized by a field-assisted dissolution model [31]. At the initiation of the process, a potential of 20 V was applied with a potential ramp of 0.2 V/s. The inset of Figure 6 presents the rapid growth of the current. After the first 10 s, when the potential reached 2 V, the current density remained at ~2 mA/cm^2^. Even the potential continued to grow; this fact indicated the oxide formation initiation process. After the potentiodynamic stage—the first 100 s, when the process arrived at the target potential of 20 V—it entered the potentiostatic stage, where the potential was kept constant. There was a sudden drop in current due to the insulator nature of the compact oxide that was growing. This evolution was interrupted by the initiation of the nanopores due to the presence of fluoride ions, which led to a rise in current. The process of growth of the nanopores/nanotubes took place during the steady state of the current when an equilibrium between oxide formation at the metal–oxide interface and oxide dissolution at the oxide–electrolyte interface was reached.

**Figure 7:**
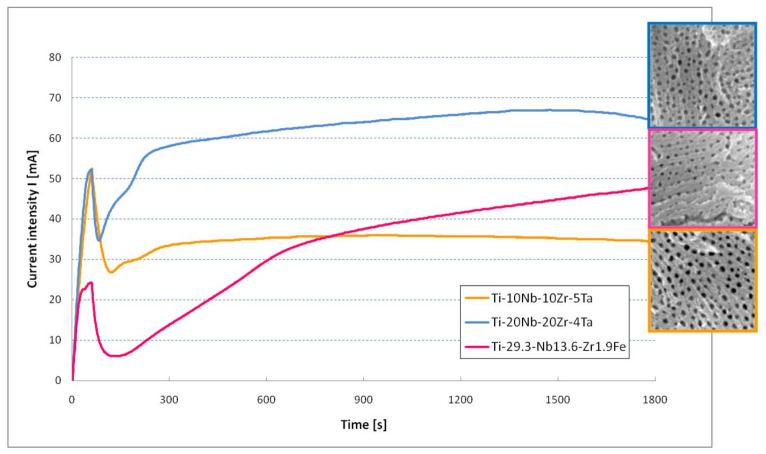
Current evolution during the EA of quaternary titanium alloys in organic electrolyte (insets: the nanostructured morphology of oxide layers).

Figure 7 shows the current evolution registered during the anodization processes in the organic electrolyte with an anodization potential of 60 V. The potential was applied with a potential ramp of 1 V/s; this reached the target value in 60 s. During this initial phase, the current increased for all three materials subjected to electrochemical anodization. As 60 V was reached, the potential was kept constant by the end of the process. Regarding the Ti-10Nb-10Zr-5Ta and Ti-20Nb-20Zr-4Ta alloys, the current intensity vs. time plots, initial oxide formation (drop in current intensity), nanopore initiation (current rise), and nanoporous structure development (steady state of current) could clearly be observed. In the case of the Ti-29.3Nb-13.6Zr-1.9Fe alloy, the steady state was not so clear, occurred later in time (after 15 min), and the current continued to slowly rise. The nanoporous morphology of the oxide was formed on the anodized surface.

## 4. Conclusions

Nanostructured oxide layers were synthesized on quaternary Ti-Nb-Zr-Ta and Ti-Nb-Zr-Fe alloys with a high-fraction of β phase by electrochemical anodization in a 1 M H_3_PO_4_ + 0.5 wt% HF aqueous electrolyte and in a 0.5 wt% NH_4_F + 2 wt% H_2_0 + ethylene glycol organic electrolyte. Regarding Ti-10Nb-10Zr-5Ta, a multicomponent oxide developed in the aqueous electrolyte at an anodization potential of 20 V with a pore/tube inner diameter of 39 ± 8.9 nm, and in the range of 35 ± 8.3–60 ± 13 nm, when anodization was performed in the organic electrolyte at 40 and 60 V, higher diameters occurring at the higher potential. The anodization of the Ti-20Nb-20Zr-4Ta alloy in the aqueous electrolyte led to nanotubular oxide development with a tube inner diameter of 43 ± 11.8 nm, with a bimodal structure where large nanotubes were surrounded by smaller ones. In the organic electrolyte, an ordered self-arranged nanoporous oxide with diameters of 31 ± 6.3 at 40 V and 60 ± 11.3 nm at 60 V was formed. On the surface of the Ti-29.3Nb-13.6Zr-1.9Fe alloy using the aqueous electrolyte, the resulting oxide morphology consisted of nanopores of 32 ± 6.8 nm diameter; by using the organic electrolyte, the nanoporous structure developed in a regular pattern, forming rows with pore openings of 38 ± 9.0 nm at 40 V and 49 ± 9.7 nm at the 60 V anodization potential.

In all cases, the oxide contained a high amount of oxygen, indicating the growth of a complex metal oxide on the sample surfaces. The presence of all alloying elements was confirmed in the oxide, together with F and C elements, which were uptaken from the electrolytes. For all samples, the most stable TiO_2_ form—anatase in a tetragonal crystalline structure and orthorhombic ZrO_2_—was detected.

The developed alloys are promising biomaterials for orthopedic applications, lowering the modulus of elasticity, in order to match that of human bone. Their surface modification to develop nanostructured oxide layers could enhance osseointegration. Such modified surfaces can act as carriers for the delivery of drugs and growth factors as well as anti-inflammatory, antibacterial, and anticancer agents and all these aspects are currently under intense research exploitation. The present research paper is integrated in this approach, and the results obtained contribute to implement the common scientific interest on this topic by recommending the process parameters for electrochemical anodization, which ensures the synthesis of a desired oxide morphology.

## Figures and Tables

**Table 1:** The values of the openings of the nanopores/nanotubes and the mass loss, depending on the EA process parameters.

Process Parameters			Material	Mass Loss, Δm (mg)	Nanostructure Diameter, D (nm)				
Electrolyte	Potential, U (V)	Time, T (min)			Average	Standard Deviation	Range	Median	Mode
1M H_3_PO_4_ + 0.5 wt% HF	20	30	Ti-10Nb-10Zr-5Ta	1.5	39	8.9	20–69	38	38
			Ti-20Nb-20Zr-4Ta	1.0	43	11.8	19–77	43	45
			Ti-29.3Nb-13.6Zr-1.9Fe	1.0	32	6.8	17–52	31	29
0.5 wt% NH_4_F + 2wt% H_2_0 + EG	40	30	Ti-10Nb-10Zr-5Ta	0.8	35	8.3	18–57	34	31
			Ti-20Nb-20Zr-4Ta	0.4	31	6.3	17–46	31	28
			Ti-29.3Nb-13.6Zr-1.9Fe	0.7	38	9.0	17–58	37	35
0.5 wt% NH_4_F + 2wt% H_2_0 + EG	60	30	Ti-10Nb-10Zr-5Ta	1.4	60	13.0	31–92	60	67
			Ti-20Nb-20Zr-4Ta	2.1	60	11.3	40–89	58	49
			Ti-29.3Nb-13.6Zr-1.9Fe	3.2	49	9.7	25–72	48	47

**Table 2:** Elemental composition of oxide layers developed by EA in different electrolytes.

	Element	Ti-10Nb-10Zr-5Ta		Ti-20Nb-20Zr-4Ta		Ti-29.3Nb-13.6Zr-1.9Fe	
	(wt%)	Whole Area	Average on Selected Areas	Whole Area	Average on Selected Areas	Whole Area	Average on Selected Areas
Aqueous Electrolyte	Ti	47.80	48.73 ± 1.21	35.30	36.20 ± 1.06	37.20	37.20 ± 1.60
	O	29.20	29.53 ± 1.16	28.80	27.67 ± 0.67	28.50	27.90 ± 0.92
	Nb	8.20	7.97 ± 0.23	14.70	15.03 ± 0.47	18.50	18.50 ± 0.64
	Zr	6.60	6.73 ± 0.21	13.40	13.67 ± 0.60	10.50	10.80 ± 0.57
	Ta	4.20	3.23 ± 0.15	3.30	2.83 ± 0.35	-	-
	Fe	-	-	-	-	1.20	1.20 ± 0.12
	F	4.00	3.77 ± 0.31	4.60	4.53 ± 0.42	4.10	4.40 ± 0.32
Organic Electrolyte	Ti	41.10	42.57 ± 0.83	32.90	32.95 ± 1.81	30.10	31.17 ± 2.27
	O	29.60	28.43 ± 0.91	29.20	28.85 ± 2.29	28.50	27.67 ± 2.00
	Nb	8.10	8.53 ± 0.15	16.50	16.08 ± 0.62	15.70	16.20 ± 0.95
	Zr	5.00	5.17 ± 0.38	10.80	10.75 ± 0.64	10.60	11.07 ± 0.91
	Ta	4.80	4.97 ± 0.15	3.30	3.93 ± 0.47	-	-
	Fe	-	-	-	-	1.00	1.00 ± 0.10
	F	7.50	6.97 ± 0.50	7.30	7.43 ± 0.56	8.20	7.93 ± 0.90
	C	3.90	3.40 ± 0.26	-	-	6.00	4.97 ± 1.29

## Data Availability

Not applicable.
